# Acetabular liner dissociation: A case report and review of the literature

**DOI:** 10.1051/sicotj/2019025

**Published:** 2019-08-28

**Authors:** Asif A.H. Parkar, Mohamed Sukeik, Ahmed El-Bakoury, James Powell

**Affiliations:** 1 Foothills Medical Centre 1403 29 St NW Calgary AB T2N 2T9 Canada; 2 University of Alexandria Egypt

**Keywords:** Liner, Dissociation, Hip, Dual mobility, Constrained implants, Arthroplasty

## Abstract

Dissociation of the polyethylene liner from the acetabular shell is a rare but catastrophic complication of total hip arthroplasty (THA). There have been reports of polyethylene liner dissociation (PLD) as well as ceramic liner dissociation (CLD) in the literature. Amongst the commonly used implants, liner dissociation has been reported with the Pinnacle (DePuy), Harris–Galante (Zimmer) and Trident (Stryker) acetabular components.

To the best of our knowledge, this is the first case report of PLD in an R3 (Smith & Nephew) acetabular component. This case report highlights the implant choice for treatment of the liner dissociation and the role of constrained implants in such cases.

## Introduction

Dissociation of the polyethylene liner from the acetabular shell is a rare but catastrophic complication of total hip arthroplasty (THA). This has been well researched and it appears that certain implant designs have shown a greater risk of polyethylene liner dissociation (PLD). Despite the excellent track record with regard to the functional outcome and longevity, Pinnacle (DePuy), Harris–Galante (Zimmer) and Trident (Stryker) have been associated with PLD [1–5].

To the best of our knowledge, this is the first case where a PLD of an R3 acetabular component (Smith & Nephew) is being reported. This is a case report of a complex THA looking at implant choice in such cases in view of the recent literature.

## Case report

**Figure 1:**
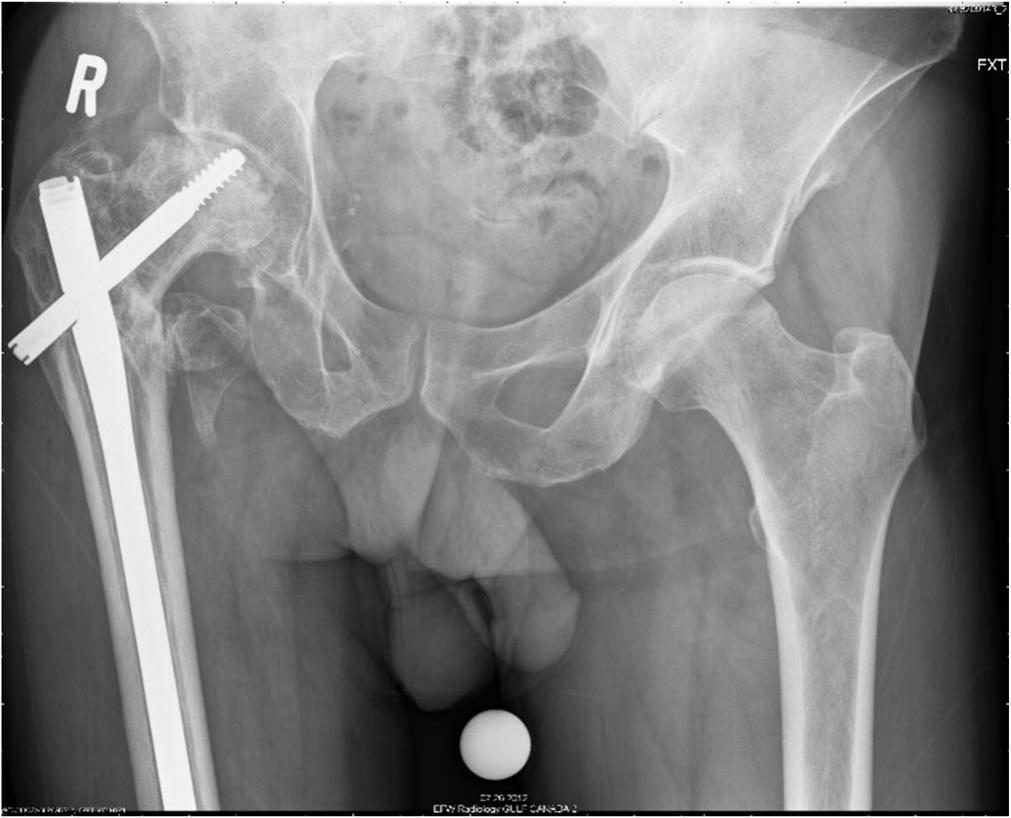
Painful right hip with collapse of the femoral head due to avascular necrosis.

A 56-year-old patient had a complex primary THA following failure of fixation of an intertrochanteric fracture treated with a Gamma nail, 7 years ago (Figure 1). His medical history included hypertension, heavy smoking, recreational drugs and excessive alcohol use.

**Figure 2:**
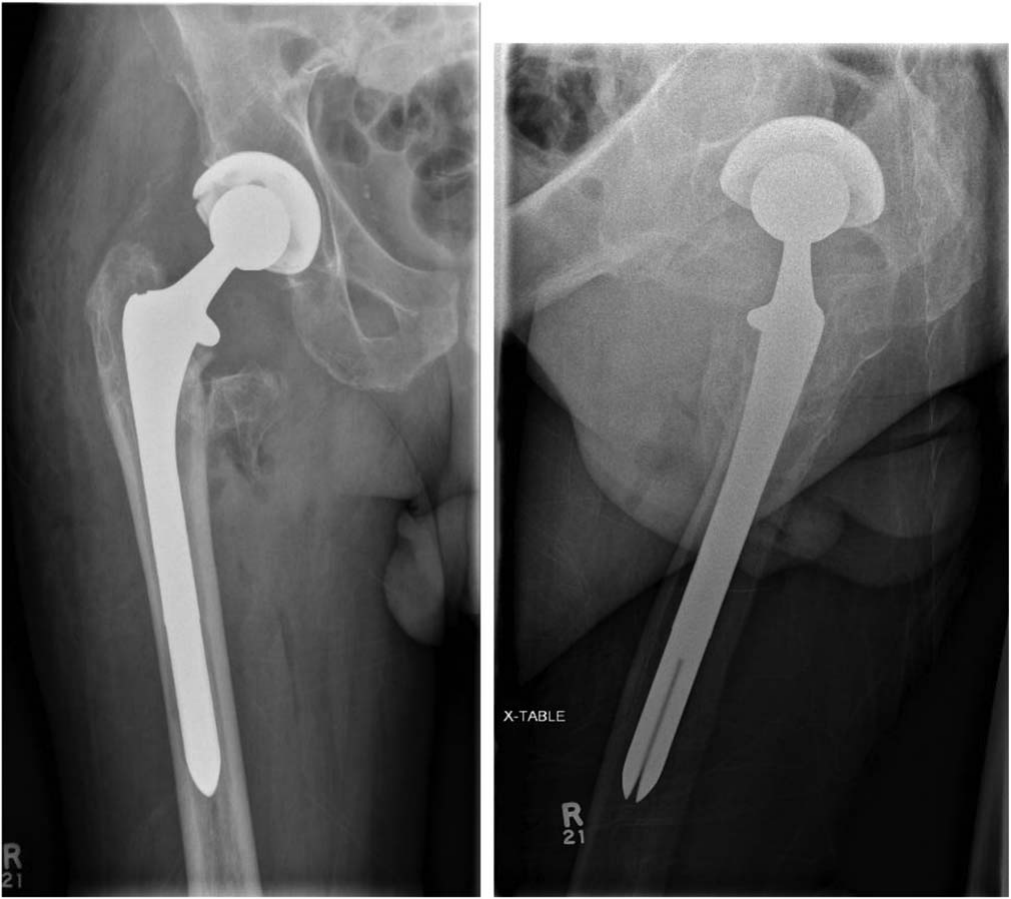
A and B: Complex primary THA.

**Figure 3:**
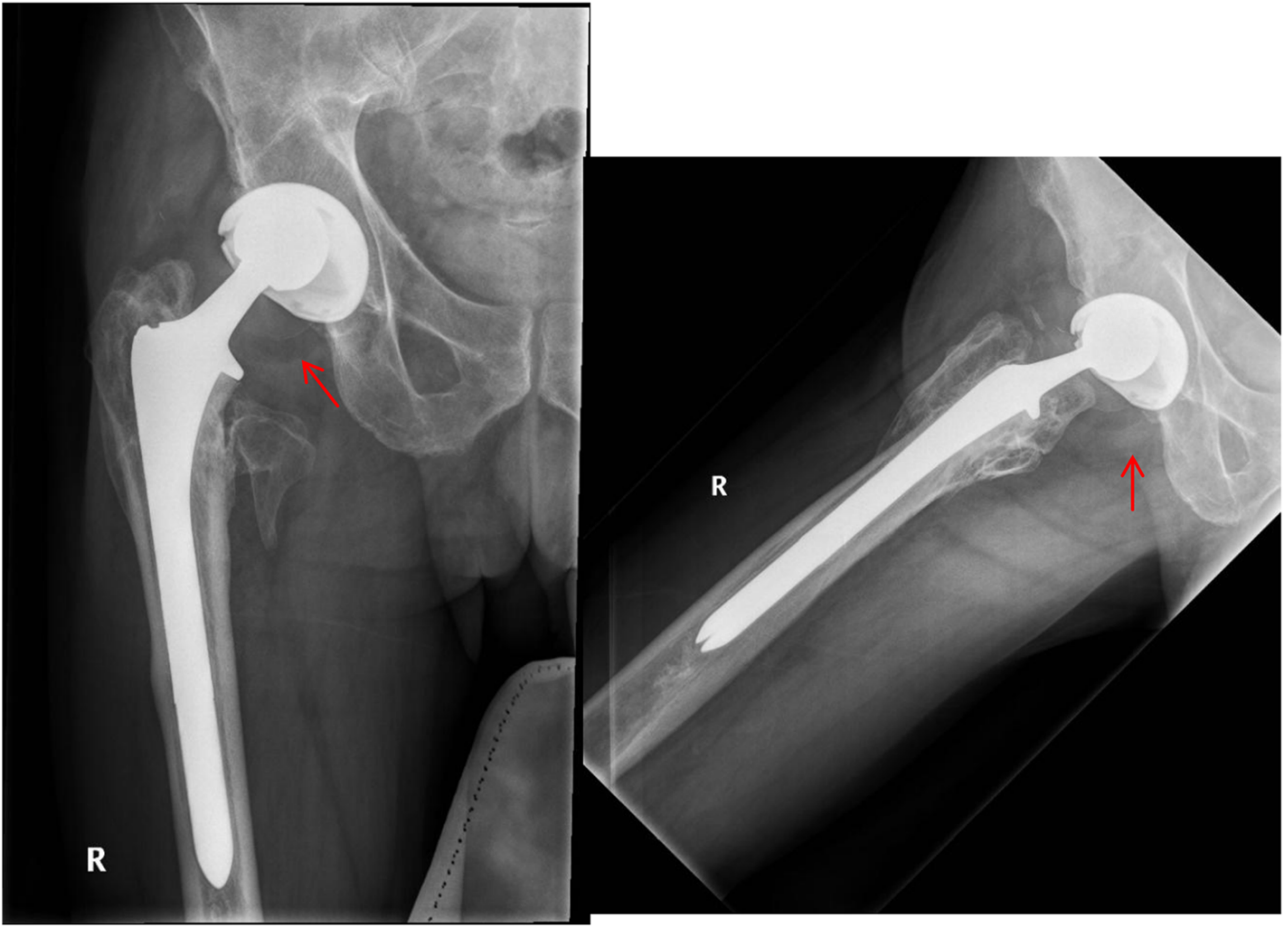
A and B: Proximal migration of the femoral head and radiolucent ring suggestive of polyethylene liner dissociation.

THA was performed after removal of the Gamma nail with extension of the old incision using a posterolateral approach. The patient required release of the gluteus maximus, iliopsoas and anterior capsule to address the flexion contractures. An uncemented THA was performed using the Smith & Nephew R3 size 60 acetabular shell with 60/36 highly cross-linked polyethylene liner in the templated position. As the old fracture was extending up to the subtrochanteric region, a size 15 Echelon uncemented stem was used to bypass the subtrochanteric region. A CoCr 36 + 10 femoral head was used and was found to be stable (Figure 2A and B). Clinical and radiographical follow up of his THA was unremarkable for 5 years postoperatively. However, 5 years after the primary operation, the patient had a fall and presented with a new onset of hip pain, and radiographs showed radiolucency medial to the femoral neck in association with an eccentrically placed femoral head lying in contact with the acetabular metal shell. Careful evaluation of the radiographs showed that this radiolucency was consistent with a PLD (Figure 3A and B).

**Figure 4:**
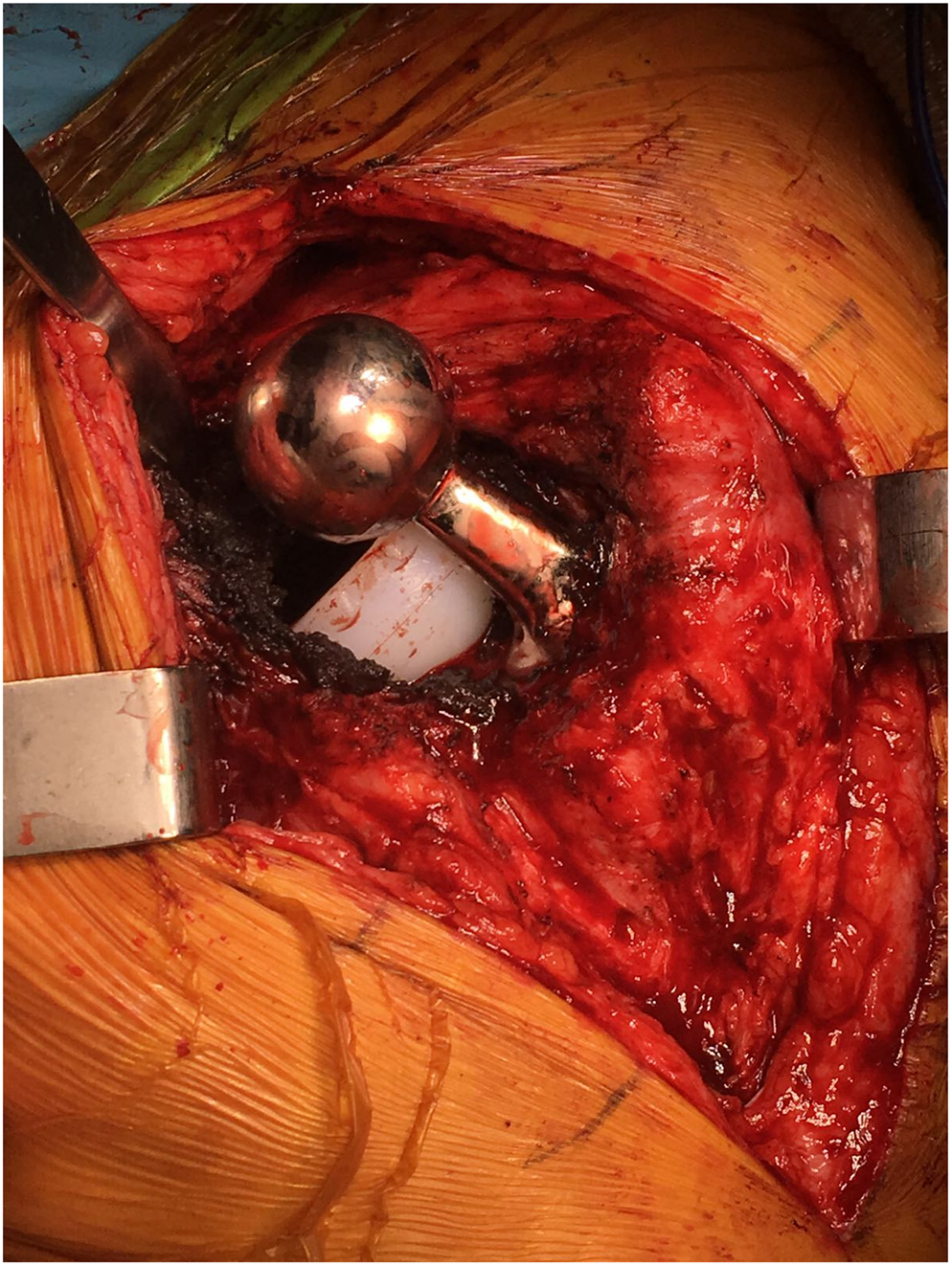
Intra operative picture showing the dissociated liner.

**Figure 5:**
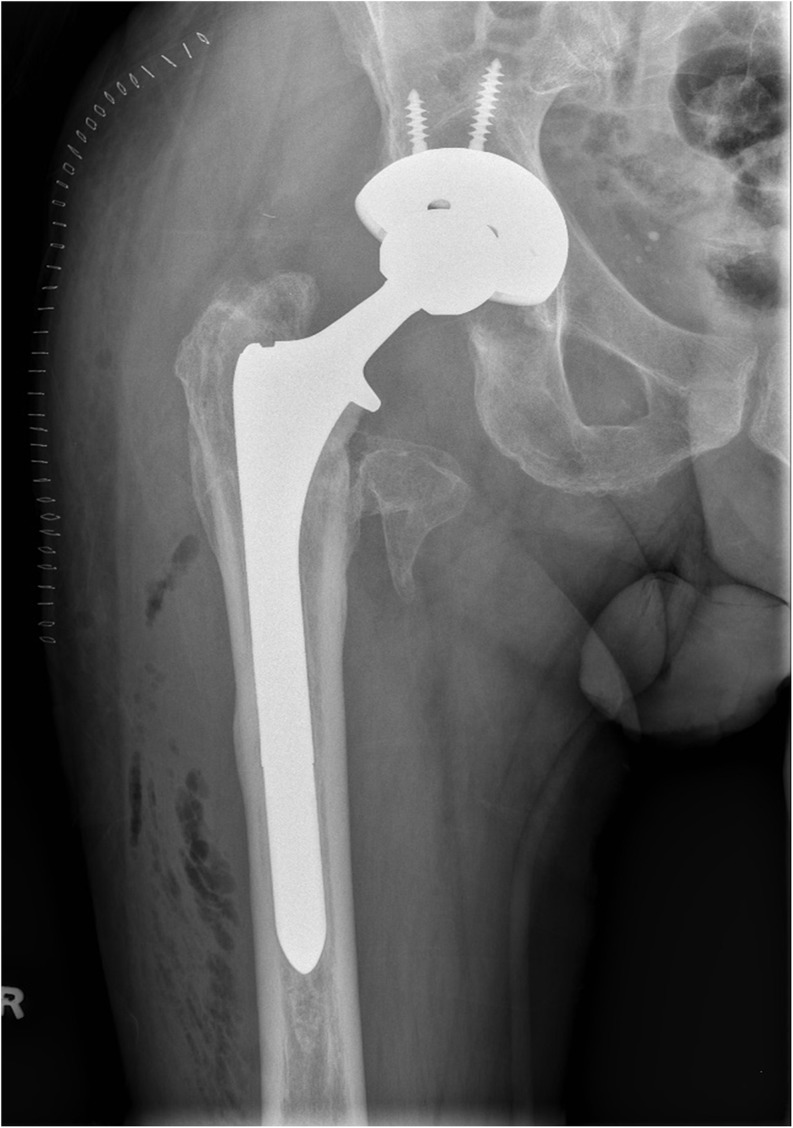
Radiographs after revision of the acetabular components.

Infection work up including inflammatory markers and a joint aspiration were clear; hence a single-stage revision was proposed. Patient declined surgery and opted to carry on for further 3 months at which point his pain worsened and hence revision arthroplasty was performed. Intraoperatively, the acetabular liner was found to be dissociated (Figure 4). The femoral component was stable and therefore retained. Surgery involved replacement of the acetabular shell and liner and the femoral head along with excision of a pseudo tumour resulting from metallosis. Trial reduction and range of movement testing were satisfactory, and hence the definitive components implanted included a Zimmer Continuum shell of size 64 mm with an offset liner and 36 mm + 12 Oxinium femoral head (Figure 5).

**Figure 6:**
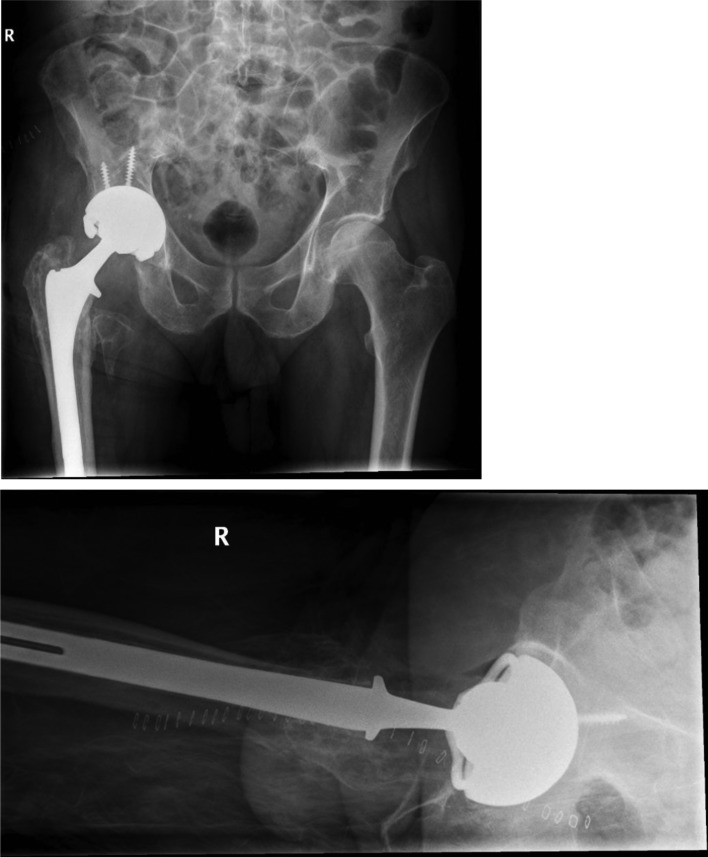
A and B: Constrained liner following the 2nd revision THA.

The patient made an unremarkable recovery in the immediate postoperative period but presented with a dislocation of his THA within a month of his operation. He underwent closed reduction under general anaesthesia but unfortunately, continued to have persistent instability, and hence a month later the patient underwent a second revision. Intraoperatively, the components were reassessed and it was confirmed that both the acetabular shell and femoral stem were well fixed in excellent position and therefore both components were retained. The polyethylene was revised and a constrained liner was used instead with a new size of 36 mm + 12 Oxinium femoral head (Figure 6A and B).

## Discussion

PLD has been described in a number of studies in the past [1–7]. The dissociation can occur either during the early or late postoperative period after a THA. Dissociations occurring in the early postoperative period are likely to be the result of inadequate seating of the polyethylene liner and failure of the locking mechanism of the acetabular component [1, 2]. Late dissociations are believed to be either from femoral neck impingement against the polyethylene liner or edge loading resulting in fatigue failure of the locking mechanism [1, 3].

The radiolucency of the dissociated liner seen on plain radiographs has been described as the “crescent” sign while using ultrasound this has been described as the “tram track” sign [8–10]. PLD has been reported with some of the most commonly used implants [1, 2, 4, 6, 11]. However, to date there have been no reports of this complication with the Smith & Nephew R3 system.

This is a case of complex arthroplasty in a patient with compliance issues due to alcoholism and substance abuse. Fixation of the femoral neck fracture failed due to avascular necrosis of the femoral head which was revised to a THA using unconstrained implants as the patient was young and this was his primary arthroplasty. A large femoral head of size 36 mm was used for better stability and the patient did well for 5 years until he had the fall which resulted in the liner dissociation. When the patient presented with the PLD, the acetabular components were revised without using any constrained options. The patient presented with a dislocated hip within a month of having the first revision for which he had a closed reduction. As he continued to have instability, this was then revised using a constrained liner, and at 1-year follow up there has been no further dislocation or any other complications.

In hindsight, we feel that considering both the patient factors and abnormal anatomy, a more constrained prosthesis might have been a better implant of choice after the patient presented with the liner dissociation. The recent literature suggests excellent survivorship with dual mobility cups and has recommended them as the primary implant of choice in high-risk patients [11, 12]. Modern dual mobility designs have resolved complications such as intra-prosthetic dislocations which were reported with the first generation [13]. Dual mobility has the advantage of addressing the head neck ratio, jump distance and the requirement for a constrained liner all at once [11]. The literature suggests that dual mobility outperforms large diameter femoral heads and constrained liners at 10 years of follow up [14].

In summary, we recommend the use of dual mobility or constrained liners as the implants of choice in complex THA cases with compromised anatomy, previous dislocation and non-compliant patients to minimise the risk of further instability.
